# A Smartphone Attention Bias Intervention for Individuals With Addictive Disorders: Protocol for a Feasibility Study

**DOI:** 10.2196/11822

**Published:** 2018-11-19

**Authors:** Melvyn Zhang, Jiangbo Ying, Syidda B Amron, Zaakira Mahreen, Guo Song, Daniel SS Fung, Helen Smith

**Affiliations:** 1 National Addictions Management Service Institute of Mental Health Singapore Singapore; 2 Department of Developmental Psychiatry Institute of Mental Health Singapore Singapore; 3 Family Medicine and Primary Care Lee Kong Chian School of Medicine Nanyang Technological University Singapore Singapore Singapore

**Keywords:** addiction, approach bias, attention bias, bias modification, feasibility, pilot, psychiatry, mobile phone, mHealth, eHealth

## Abstract

**Background:**

Substance use disorders are highly prevalent globally. Relapse rates following conventional psychological interventions for substance use disorders remain high. Recent reviews have highlighted attentional and approach or avoidance biases to be responsible for multiple relapses. Other studies have reported the efficacy of interventions to modify biases. With advances in technologies, there are now mobile versions of conventional bias modification interventions. However, to date, no study has evaluated bias modification in a substance-using, non-Western sample. Existing evaluations of mobile technologies for the delivery of bias interventions are also limited to alcohol or tobacco use disorders.

**Objective:**

This study aims to examine the feasibility of mobile-based attention bias modification intervention among treatment-seeking individuals with substance use and alcohol use disorders.

**Methods:**

This is a feasibility study, in which inpatients who are in their rehabilitation phase of clinical management will be recruited. On each day that they are in the study, they will be required to complete a craving visual analogue scale and undertake both a visual probe-based assessment and and modification task in a smartphone app . Reaction time data will be collated for the computation of baseline attentional biases and to determine whether there is a reduction of attentional bias across the interventions. Feasibility will be determined by the number of participants recruited and participants’ adherence to the planned interventions up until the completion of their rehabilitation program and by the ability of the app in detecting baseline biases and changes in biases. Acceptability of the intervention will be assessed by a short questionnaire of users’ perceptions of the intervention. Statistical analyses will be performed using SPSS version 22.0, while qualitative analysis of the perspectives will be performed using NVivo version 10.0.

**Results:**

This study was approved by the National Healthcare Group Domain Specific Research Board, with approval number (2018/00316). Results will be disseminated by means of conferences and publications.Currently, we are in the process of recruitment for this study.

**Conclusions:**

To the best of our knowledge, this is the first study to evaluate the feasibility and acceptability of a mobile attention bias modification intervention for individuals with substance use disorders. The data pertaining to the feasibility and acceptability are undoubtedly crucial because they imply the potential use of mobile technologies in retraining attentional biases among inpatients admitted for medical-assisted detoxification and rehabilitation. Participants’ feedback pertaining to the ease of use, interactivity, and motivation to continue using the app is crucial because it will determine whether a codesign approach might be warranted to design an app that is acceptable for participants and that participants themselves would be motivated to use.

**International Registered Report Identifier (IRRID):**

PRR1-10.2196/11822

## Introduction

### Background

Illicit drug and alcohol use are highly prevalent globally. The United Nations Office on Drugs and Crime reported that in 2015, a quarter of a billion individuals experimented with substances, and 29.5 million individuals were diagnosed with substance disorders [1]. Cannabis, opioids, and stimulants such as amphetamines are most commonly abused [1]. Substance use is associated with other comorbidities like retroviral diseases and hepatitis C infection. In addition, substance use is associated with significant mortality, and in 2015, an estimated 190,000 deaths were attributed to drug use [1]. Globally, alcohol use is a major problem too [2]. Reportedly, alcohol disorders tend to be more prevalent in developed countries and higher-income status countries [2]. In the most recent report released by the World Health Organization, it was reported that the harmful use of alcohol resulted in an overall mortality of 3 million individuals in 2016 [3]. In Singapore, in a prior study conducted by the World Health Organization in 2004, the prevalence of alcohol use disorders among females and males was estimated to be 0.19% and 1.40%, respectively, and the prevalence of drug use disorders among females and males to be 0.07% and 0.28%, respectively [4]. While there are no large-scale prevalence studies in Singapore, the Central Narcotics Bureau has reported that in 2017, 40% of those apprehended were new abusers, and >64% of these new abusers were under the age of 30 years [5]. Of 3091 abusers arrested in 2017, 1991 were abusing methamphetamine, and the remaining 848 and 204 were abusing heroin and cannabis, respectively. Of importance, 2570 abusers were polysubstance abusers. Thus, substance use disorders are of growing concern as indicated by the increasing numbers of offenders and their changing demographics. Taking into consideration the prevalence, there is a growing need for efficacious interventions.

The treatment options for substance use are both pharmacological and nonpharmacological. Pharmacological options are varied; for opioid use disorders, opiate substitution therapy such as that with methadone or buprenorphine could be considered. In Singapore, opiate substitution therapy is only prescribed for elderly individuals and pregnant women [6] as legislation restricts its administration for other patient groups. The prescription of opiate substitution medications such as buprenorphine is limited due to the risk of diversion [7] and medical comorbidities like infective endocarditis and other cutaneous complications [8,9]. For cannabis and amphetamine disorders, pharmacological options are limited, and symptomatic medications such as hypnotics or antipsychotics are considered in the acute treatment phase. Given the limited pharmacological therapeutic options, especially with the absence of medications to help with abstinence, psychological options are integral. Therapies like cognitive behavioral therapy are effective, with an effect size of 0.45 (Cohen *d*) [10]. Nonetheless, despite this effectiveness, 40%-50% of individuals relapse within a year of successful treatment and another 70% relapse within 3 years [11]. Such high relapse rates following a moderately effective intervention demonstrate that conventional psychological therapies may not be adequately addressing the factors leading individuals to a lapse or relapse.

Recent advances in experimental psychology have reported how automatic attentional biases could predispose individuals toward a relapse. Field et al [12] and Cox et al [13] have highlighted the presence of these automatic attentional processes in individuals with substance use disorders and recommended the use of interventions to modify these automatic processes. Attentional biases refer to the preferential allocation of attention toward substance-related stimuli [14,15], while approach biases refer to automatic tendencies to reach out and approach substance-related cues [16]. The underlying theoretical basis is that of the dual-process model, which suggests that the repeated use of a substance would lead to increased automatic processing of the substance-related cue and, hence, increased automatic tendencies to approach substance-specific cues and corresponding inhibition of normal cognitive control processes. Tasks such as the visual probe task have been used for bias assessment and modification. In the conventional visual probe task, a pair of images is presented, in which one is substance related and the other is a neutral image. Following the presentation of the images, a small probe (or stimulus such as an arrow) would replace one of these images. Participants are required to indicate the position of the stimulus or the direction of the arrow as quickly as they could. When the visual probe task is used for bias modification, the task is as described, but the frequencies at which the probe or stimulus replace the drug or neutral image is altered [14].

There has been an extensive evaluation of bias modification for substance use disorders, and a recent meta-analysis study synthesized the overall effectiveness of bias modification [14]. Cristea et al [14] were only able to identify trials relating to alcohol or tobacco use disorders (25 trials). They reported that bias modification for both attentional and approach biases was moderately effective with an effect size of 0.60 (Hedge 0.60) [14]. However, a prior review found no association between the reduction in biases and other outcomes such as cravings, and the authors posited that this might be because more time is needed for a change in biases to be reflected regarding a change in symptomatology [14]. Nevertheless, a prior review demonstrated that bias modification could potentially reduce biases. Since the publication of Cristea et al’s [14] meta-analyses, there have been other publications [17] highlighting the problems with the evidence synthesized, given that the evidence synthesis included both experimental laboratory studies and clinical studies. Wiers et al [17] reviewed the evidence by the nature of studies and reported small and robust effects of bias modification on treatment outcome when biases were retrained in a clinical setting for individuals with alcohol use disorders. Since Cristea et al’s meta-analysis [14], Zhang et al [18] conducted a systematic review and reported that attentional biases were present in opioid use and stimulant use disorders. Other studies have evaluated attention bias modification among substance-using individuals. Ziaee et al [19] recruited a sample of opioid users who were on methadone maintenance and found that attentional bias modification led to a reduction in attentional biases, as well as cravings to use, dosing of medications, and relapses. Wolf et al [20] recruited a sample of moderate and heavy cannabis-using students and found an attentional bias only for heavy users of cannabis and not moderate users; the authors did not find bias retraining to be effective. Mayer et al [21] undertook the first study evaluating the efficacy of bias modification for treatment-seeking individuals with cocaine use disorders. Like Wolf et al [20], Mayer et al [21] also reported that bias modification was not effective. Not all studies have examined treatment-seeking individuals in a clinical setting. Schoenmakers et al [22] administered attentional bias modification to 43 individuals with alcohol dependence and reported that bias modification was effective in increasing individuals’ ability to disengage from alcohol cues. Manning et al [23] stressed the importance of evaluating a clinical sample that was in a detoxification and rehabilitation program because intervening during this period could capitalize on neural recovery. To date, Wiers et al [24], Eberl et al [25], and Manning et al [23] have examined bias modification among individuals admitted for medical-assisted detoxification and rehabilitation. While these studies have provided evidence for the effectiveness of bias modification during this critical period, the biases targeted were approach or avoidance biases and the evaluation was limited to individuals with alcohol disorders. To date, no study has evaluated attention bias modiifcation in a non-Western sample among a group of substance-using individuals who are in treatment (medical-assisted detoxification or rehabilitation).

Advances in technologies in the last decade have transformed how bias modification interventions are delivered. An increase in the number of remote Web-based therapies has been attributed to the advances in electronic health (eHealth). eHealth technologies facilitate the delivery of low-cost psychotherapy, which is highly accessible and enables anonymity of use [26]. This has led to increasing research examining the effectiveness of Web-based interventions. Further advances in technologies coupled with increased ownership of mobile devices have resulted in a growing number of interventions that tap on mobile technologies. Mobile health (mHealth) technologies are increasingly being harnessed for the delivery of bias modification as mobile technologies allow for training to be conducted in diverse locations, thus, helping in the generalization of clinical benefits [27]. The use of mobile technologies enables the frequency of training to be increased, which may improve outcomes [27]. mHealth technologies have an advantage over existing Web-based version as they do not require individuals to be consistently connected to the internet to undertake the bias modification task. Zhang et al [28] in their review managed to identify 8 studies that have evaluated the potential and effectiveness of mHealth bias modification. Seven studies [29-35] reported that mHealth bias modification was effective; these studies were conducted among participants with insomnia, alcohol, tobacco use, or social anxiety disorders. Only a single study reported mobile bias modification to be ineffective. Despite the evidence for effectiveness being inconclusive, Zhang et al’s [28] prior review reported bias modification to be effective for addictive disorders such as alcohol and tobacco use disorders.

### Rationale for This Study

To date, no study has evaluated bias modification in a substance-using, non-Western, treatment-seeking sample. In addition, while technologies like eHealth and mHealth have been widely utilized for the delivery of bias modification interventions, our prior review [28] of the published literature demonstrated that mobile technologies have only been evaluated among individuals with alcohol or tobacco use disorders. Hence, this proposed study aims to examine the feasibility of a mobile-based attention bias modification intervention among treatment-seeking individuals with alcohol or substance use disorders. If deemed feasible, this will guide further evaluative research investigating the efficacy of such a complex intervention.

### Objectives

The primary aim of this study is to determine the feasibility of an attention bias modification mobile app for the reduction of attention biases to substance-related cures among individuals with addictive disorders. Feasibility is determined by the number of participants recruited, participants’ adherence to the planned interventions up until the day of discharge, and the ability of the app to detect baseline biases and changes in biases. The secondary aim is to determine the acceptability of the intervention, which will be assessed through a questionnaire of users’ perceptions.

### Research Questions

1. Will the mobile attention bias modification intervention be feasible and acceptable among individuals with addictive disorders?
2. Is the developed mHealth app capable of detecting the changes in biases?

## Methods

### Study Setting and Study Design

This study will be conducted among individuals who are admitted for inpatient medication-assisted detoxification and rehabilitation at the National Addictions Management Service (NAMS), Institute of Mental Health Singapore. Notably, at NAMS, all patients are admitted voluntarily for treatment, which implies that patients are free to discharge should they be not willing to complete the detoxification or rehabilitation phase of the program. At any one time, a maximum of 30 patients could be accommodated in the ward environment. Patients are managed by an attending psychiatrist with a multidisciplinary team comprising addiction-trained counselors, nurses, and social workers. In the first week of the treatment program, patients would undergo medication-assisted detoxification. In the second week of their treatment program, patients would attend community meetings and group-based counseling. Individual counseling will also be provided to patients. Only participants who have completed their detoxification phase (first week of their stay) will be recruited for this study as participants would be free from withdrawal symptoms. This is a feasibility study, where participants’ attention biases will be assessed following the bias modification intervention. The study protocol has been approved by the National Healthcare Group’s Domain Specific Research Board (May 2, 2018; Ethics Approval Reference Number: 2018/00316).

### Recruitment

All participants will be recruited from the inpatient unit at the NAMS, Institute of Mental Health, Singapore. Participants will be informed of the study by their attending health care professionals on admission and will be approached by the study team on the first day of their rehabilitation phase. The study team will provide participants with further information about the study, and if participants agree to participate, they will complete the informed consent form.

### Sample Size

Power computation has not been undertaken for this study as study design is that of a feasibility study. Considering the diversity of the addictive disorders included, the proposed sample size is 30 participants.

### Eligibility Criteria

#### Inclusion and Exclusion Criteria

Participants will be eligible for the feasibility study if they meet the inclusion criteria presented in Textbox 1.

Participants will not be eligible if they meet any of the exclusion criteria presented in Textbox 2.

Participants with moderate to severe symptoms of comorbid psychiatric disorders are excluded from this feasibility study as there is a high likelihood that their comorbid psychiatric disorder will affect their attentional bias scores. Moreover, patients with other psychiatric disorders will usually be on other psychotropic medications. Zhang et al have highlighted that several pharmacological agents, such as those targeting dopaminergic, noradrenaline, glutaminergic, and serotonergic neurotransmissions, have an acute effect on attentional biases [36].

### Intervention

Patients will be invited to participate in this study only upon the completion of their medical detoxification treatment. If they consent to participate, they will complete a set of baseline questionnaires including a demographic and clinical information questionnaire, the Addiction Severity Index (ASI)-Lite questionnaire, the Severity of Substance Dependence (SDS) questionnaire, and the Short-Form 12 (SF-12) questionnaire. In addition, participants are required to complete a visual analogue scale for craving before and after the completion of each session. Table 1 provides an overview of the outcome measures that participants need to complete for each session. Participants will receive a 15-minute briefing on the use of the mobile app (by members of the study team who are familiar with the app) before the commencement of the assessment and intervention. Participants will be provided with tablets by the study team to use the mobile attention bias modification intervention.

**Inclusion criteria.**

- Participants aged 21-65 years
- Participants diagnosed with a primary psychiatric disorder of alcohol dependence, opioid dependence, cannabis dependence, stimulant dependence, or polysubstance use disorder
- For participants diagnosed with polysubstance use disorder, the main substance of use must be alcohol, opioid, cannabis, or a stimulant
- Participants need to be able to read and write in English
- Participants need to be able to use a smartphone or a tablet device

Exclusion criteria

- Having a known history of cognitive impairment or dementia
- Having a history of seizures (apart from febrile seizures) or a prior history of withdrawal seizures
- Having a medical history of migraines (triggered by flashing lights)
- Having moderate to severe symptoms of comorbid psychiatric disorders (affective disorders, anxiety disorders, and psychotic disorders) based on clinical assessment

**Table 1:** An overview of the outcome measures that participants need to complete for each session.

Instrument	Aim	Day of rehabilitation				
		1	2	3	4	5^a^
Baseline demographic and clinical information questionnaire	Baseline characteristic of participants	✓				
Addiction Severity Index questionnaire	Details about substance use	✓				
Severity of Substance Dependence questionnaire	Assessment of severity of psychological dependence	✓				
Short-Form 12	Baseline health status	✓				
Attention bias modification assessment task	Measurement of attentional biases	✓^b^	✓	✓	✓	✓
Attention bias modification intervention task	Retraining of attentional biases	✓	✓	✓	✓	✓
Visual analogue scale for craving	Assessment of cravings	✓^c^	✓^c^	✓^c^	✓^c^	✓^c^
Perspective questionnaire	Acceptability of intervention			✓		

^a^Participants will undertake a maximum of 5 sessions considering that the study will not be conducted on weekends.

^b^The attention bias modification assessment task will be completed twice on the first day: first assessment will provide information pertaining to the baseline attentional biases; second assessment will assess for the change in attentional biases following the first intervention.

^c^Start and end of each session.

On the first day of their rehabilitation, participants will be required to complete a baseline attention bias assessment task and an attention bias modification task. They will be allowed to rest for 15 minutes before they complete a reassessment of their attention bias. Participants can rest for slightly longer on the first day, given that they are required to complete two tasks—an assessment and a modification task. On the subsequent days of their rehabilitation, they will complete the attention bias modification task and will be allowed 10 minutes of rest before retaking an attention bias assessment task. Participants will be required to complete the visual analogue scale for craving before and after the completion of each of the bias modification task. Participants who have completed 3 sessions will be asked to complete the app perception questionnaire. Participants are expected to undertake the intervention throughout their rehabilitation stay on the ward, except for weekends and public holidays. The intervention is not administered on these days due to the possibility of image-triggered cravings and the absence of a counselor to address the heightened cravings.

**Figure 1:**
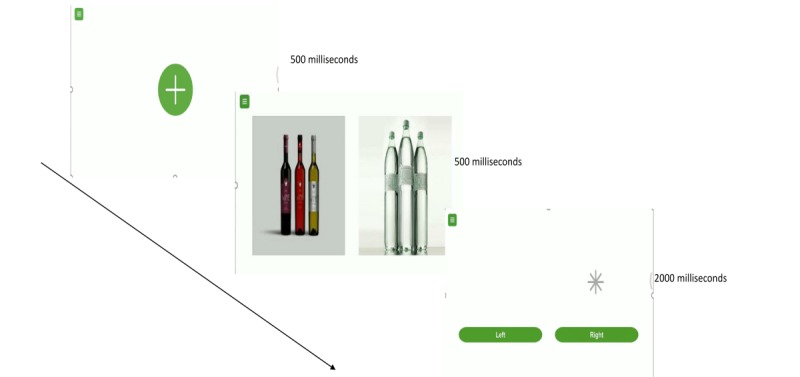
Overview of the task that participants undertake on the smartphone or tablet device.

The mobile version of the visual probe task that participants will use adheres to the protocol of the original visual probe task [37]. In the attention bias assessment task, participants will be required to complete a total of 200 trials (with 10 set of images being repeated 20 times). In each trial, participants will be presented with a fixation cross in the center of the screen for 500 ms. Subsequently, they will be presented with a set of two images for another 500 ms. In each set of images, one of the images will be a neutral image, which is closely related to that of the alcohol or drug image (eg, an image of a man drinking from a can of beer, which will be paired with tan image of a man drinking from a soft drink can). Following the disappearance of the images, an asterisk will replace the position of one of the images (either on the right or on the left). Participants will be required to indicate a response by selecting the physical onscreen buttons as fast as they could. The next set of images will be presented once participants have indicated a response (by pressing the left or right button depending on where the probe is) or if the time of 2000 ms has lapsed (Figure 1). In the assessment phase, 50% of the time, the asterisk will replace the neutral image, and the rest 50% of the time, it will replace the alcohol or substance image. For the intervention or bias modification task, participants will be required to take the same task as that described. However, the asterisk will replace the position of the neutral image 100% of the time for the attentional bias to be retrained. Figure 1 provides an overview of the task that participants undertake on the smartphone or tablet device.

### Outcomes

The primary outcome feasibility is defined by the number of participants recruited and participants’ adherence to the intervention. In our protocol, we have proposed a total recruitment sample size of 30 participants, and the study is considered feasible if we manage to recruit 25% of the invited number of participants (8 participants). We have proposed a 25% recruitment target for feasibility as we anticipate that there will be challenges to recruitment as the inpatient treatment program is a voluntary program (ie, patients could be discharged voluntarily from the program at any time) and that some patients would leave following the completion of their medical-assisted detoxification. In addition, 25% has been proposed as the target, due also, in part, to our strict inclusion and exclusion criteria. We have specifically mentioned that we will exclude participants with medical comorbidities such as seizures and migraines. To a large extent, most individuals with alcohol disorders often have a history of withdrawal seizures and, hence, they are not eligible for the study, thus, potentially affecting the overall recruitment. Furthermore, we have specifically mentioned that individuals with moderate to severe psychiatric comorbidities are not included. As other psychiatric conditions like depression or anxiety disorders are often comorbidities of substance use disorder, a large group of individuals cannot be recruited. The other criterion for feasibility is patients’ adherence to the planned interventions, and we will consider the study to be feasible if all participants complete at least 60% of the planned interventions.

Attention bias will be computed using the mean reaction time that participants take to react to asterisks or probes that replace the neutral and alcohol or substance images in the assessment task. Attention bias is present if the mean reaction time taken by participants to react to probes that replace the neutral image is longer in comparison to that of alcohol or substance images. The underlying rationale is that an alcohol or substance image would preferentially draw one’s attention, and the reaction time taken to react to asterisk or probes replacing the image is naturally much faster. Attention bias, as measured by the assessment tasks, will be compared to determine if the interventions result in a reduction in overall biases (by comparing the final attention bias score with the baseline score). Cravings will be assessed using the single-item measure, the visual analogue scale [38], with scores ranging from 0 (no cravings) to 100 (extreme levels of cravings). The visual analogue scale is often used for the assessment of current cravings and urges mainly in terms of craving frequency and intensity [30]. There are inherent advantages to the use of the single-item visual analogue scale given that it is easy to administer [30], and in our case, it would also help reduce respondents’ burden and risk of refusal, given the need for repeated measures. The scores on the visual analogue scales will be compared before and after the sessions and between sessions. If participants report increased cravings, they will be referred for counseling.

The secondary outcome acceptability will be assessed through a perception questionnaire, which consists of the following questions:

1. Prior to using the app, how confident are you in managing your addiction problems? (rated on a 5-point Likert scale)
2. How easy was it to use the app? (rated on a 5-point Likert scale and participants are asked to provide verbatim comments)
3. How interactive was the app? (rated on a 5-point Likert scale and participants are asked to provide verbatim comments)
4. Do you feel motivated to continue using the app? (rated on a 5-point Likert scale and participants are asked to provide verbatim comments)
5. Do the images in the app remind you of your substance use? (rated on a 5-point Likert scale)
6. After using the app, how confident are you in managing your addiction problem? (rated on a 5-point Likert scale).

The study is deemed acceptable if participants are willing to use the app daily; if at least 30% of participants rate ease of use, interactivity, motivation, and reality (questions 2-5) positively (either very or extremely on the 5-point Likert scale); and if at least 30% of participants perceive a change in their confidence level after having received 3 sessions of the intervention task (questions 1 and 6). The app is also deemed acceptable by the absence of any severe adverse events (such as intense cravings leading to a premature discharge from the inpatient program).

Baseline demographic and clinical information will be collected from participants. This includes information about nationality, gender, martial status, race, religion, highest level of employment, housing conditions, current substance use, method of consumption of substance, quantity of substance consumed each time, frequency of use, previous treatment history, chronic diseases, (psychiatric or physical disorders), and current psychiatric medications. Furthermore, participants are required to complete a modified ASI-Lite, SDS, and SF-12 questionnaires.

The ASI-Lite collates information for the following domains: drug and alcohol use, medical, employment or school, legal, family, and social and psychiatric [39]. In our modified version, we have retained the drug and alcohol use questions. Participants will be asked about their alcohol and substance use in the last 30 days, last month, and their lifetime use. The SDS comprises 5 items, all of which are explicitly concerned with the psychological components of dependence [40]. The scale has been used to measure the degree of dependence among individuals using different types of substances [40]. The SF-12has been widely used in the assessment of self-reported quality of life. It covers the 8 health domains as the original SF-36 [41].

### Data Management and Monitoring

All participants will be allocated a subject number upon recruitment. No participant-related identifiers will be captured on the hard copy forms. The completed hard copy forms and questionnaires will be stored in secure, locked cabinets in a restricted area. The electronic data from the smartphone app will be automatically synchronized onto a secured, password-protected cloud database. The main investigator will back-up a copy of the electronic data records onto a local secured computer. The principal investigator and the research assistants will take the responsibility for coding the data from the hard copy forms. An independent coinvestigator will routinely check the data entry for reliability and quality. All records will be kept securely for at least 6 years after the completion of the study.

### Planned Statistical Analyses

Data collated with be analyzed using SPSS version 22 (SPSS Inc, IL, USA). Baseline demographic information of subjects will be summarized using descriptive statistics, including means and SDs. Chi-square tests or Fisher’s test will be considered to examine any differences in the baseline demographic characteristics among the different substance disorders. The presence of attentional biases will be determined based on the mean reaction times taken to respond to the position of the probes that replace drug or neutral stimuli. In addition, statistical tests (analysis of variance) will be performed to determine whether there are statistically significant differences in the attentional bias scores across the sessions for participants. *P*<.05 will be considered statistically significant. Participants’ perspectives and feedback will be analyzed qualitatively using a thematic analysis approach using NVivo version 10.0.

### Adverse Events

Any adverse events that occur during the conduct of the feasibility study will be reported to the Domain Specific Research Board in accordance with the local institutional policy.

### Dissemination Policies

We will publish our research in peer-reviewed journals and will also present the findings at regional and international conferences.

### Patient and Public Involvement

Patients and public were not involved in planning for this protocol.

## Results

This study was approved by the National Healthcare Group Domain Specific Research Board, with approval number (2018/00316). Results will be disseminated by means of conferences and publications. Currently, we are in the process of recruitment for this study.

## Discussion

To the best of our knowledge, this is the first study to evaluate the feasibility and acceptability of a mobile attention bias modification intervention for individuals with substance use disorders such as cannabis opioid, stimulant, and alcohol disorders. The data pertaining to the feasibility and acceptability are undoubtedly crucial because they imply the potential use of mobile technologies in retraining attentional biases among inpatients who are admitted for medical-assisted detoxification and rehabilitation. Participants’ feedback pertaining to the ease of use, interactivity, and motivation to continue using the app is crucial because it will determine whether a codesign approach might be warranted to design an app that is acceptable for participants and that participants themselves would be motivated to use. Apart from substance use disorders, increasing research has demonstrated the presence of attentional biases in behavioral disorders such as gambling and internet gaming disorders [42,43]. If deemed feasible, attention bias modification could potentially be applicable to behavioral disorders, and the use of mobile technologies would be most appropriate for low-resource settings [44]. Moreover, prior meta-analysis research has demonstrated that behavioral forms of addiction, such as internet addiction, are positively associated with alcohol abuse [45]. Hence, attentional biases appear to be a common therapeutic target in both these conditions.

## Abbreviations

ASI: Addiction Severity Index
eHealth: electronic health
mHealth: mobile health
NAMS: National Addictions Management Service
SDS: Severity of Substance Dependence
SF-12: Short Form-12
